# Autism Spectrum Disorder Diagnostic System Using HOS Bispectrum with EEG Signals

**DOI:** 10.3390/ijerph17030971

**Published:** 2020-02-04

**Authors:** The-Hanh Pham, Jahmunah Vicnesh, Joel Koh En Wei, Shu Lih Oh, N. Arunkumar, Enas. W. Abdulhay, Edward J. Ciaccio, U. Rajendra Acharya

**Affiliations:** 1 School of Engineering, Ngee Ann Polytechnic, 535 Clementi Rd, Singapore 599489, Singapore; PHAM_The_Hanh@np.edu.sg (T.-H.P.); e0145834@u.nus.edu (J.V.); falco_peregrinus14@yahoo.co.uk (J.K.E.W.); shulih@hotmail.com (S.L.O.); 2 Department of Electronics and Instrumentation, SASTRA University, Thirumalaisamudram, Thanjavur 613401, India; arun.nura@gmail.com; 3 Biomedical Engineering Department, Faculty of Engineering, Jordan University of Science and Technology, P.O.Box 3030, Irbid 22110, Jordan; ewabdulhay@just.edu.jo; 4 Department of Medicine – Columbia University New York, 630 W 168th St, New York, NY 10032, USA; edwardciaccio@gmail.com; 5 Department of Bioinformatics and Medical Engineering, Asia University, 500, Lioufeng Rd., Wufeng, Taichung 41354, Taiwan; 6 International Research Organization for Advanced Science and Technology (IROAST) Kumamoto University, Kumamoto, 2-39-1 Kurokami Chuo-ku, Kumamoto 860-855, Japan

**Keywords:** autism spectrum disorder, computer-aided brain diagnostic system, EEG signals, higher-order spectra bispectrum, nonlinear features, locality sensitivity discriminant analysis, *t*-test, classifiers, 10-fold validation

## Abstract

Autistic individuals often have difficulties expressing or controlling emotions and have poor eye contact, among other symptoms. The prevalence of autism is increasing globally, posing a need to address this concern. Current diagnostic systems have particular limitations; hence, some individuals go undiagnosed or the diagnosis is delayed. In this study, an effective autism diagnostic system using electroencephalogram (EEG) signals, which are generated from electrical activity in the brain, was developed and characterized. The pre-processed signals were converted to two-dimensional images using the higher-order spectra (HOS) bispectrum. Nonlinear features were extracted thereafter, and then reduced using locality sensitivity discriminant analysis (LSDA). Significant features were selected from the condensed feature set using Student’s *t*-test, and were then input to different classifiers. The probabilistic neural network (PNN) classifier achieved the highest accuracy of 98.70% with just five features. Ten-fold cross-validation was employed to evaluate the performance of the classifier. It was shown that the developed system can be useful as a decision support tool to assist healthcare professionals in diagnosing autism.

## 1. Introduction

A shortfall in social interaction and nonverbal communication emerging as early as the first three years of life is recognized as autism spectrum disorder (ASD). ASD is a multifactorial neurodevelopment disorder that stems from genetic or non-genetic factors [1]. The etiology of ASD includes genes such as EN2(Engrailed 2) [2], UBE3A (Ubiquitin protein ligase E3A) locus, GABA (Gamma-aminobutyric acid) system, and serotonin transporter [3], which have been found to be linked to cerebellar development. Some environmental factors such as low birth weight, unusually short gestation period, viral infections, hypoxia, harm by mercury, and maternal diabetes are believed to contribute to ASD in young children [4,5]. Poor eye contact; grappling with expressing, controlling, or understanding emotions; intensified focus on a single thing; delayed speech; and social withdrawal are some tell-tale signs of ASD [6]. About 1 in 160 children are diagnosed with ASD [6] and the prevalence has heightened in the past 20 years [7]. The possibility of female genes exhibiting particular protective effects against autistic impairments [8] may be suggestive of ASD affecting males primarily [9,10] as compared to females. At present, the gold standard for autism detection includes the assessment of behavioral, historical, and parent-report information by a team of experts. However, this process is long-winded [11]; hence, diagnosis at an early stage may be delayed. The breakthrough in neuroimaging modalities such as magnetic resonance imaging (MRI) has led to the discovery that the amygdala is a main part of the brain related to the onset of autism [12]. Howard et al. [13] reported the rise in bilateral amygdala volume as well as a decrease in hippocampal and para hippocampal and gyrus volumes in ASD patients in an MRI study. In a voxel-based whole-brain examination study, Abel et al. [14] reported an increase in left amygdala volume, as well as in the right inferior and middle temporal gyruses. However, these techniques exhibit some disadvantages. MRI scans are expensive as compared to computed tomography (CT) scans [15]. Yet, CT scans and positron emission tomography (PET) are prone to analysis error due to artifacts produced by head motion [16]. A cost-effective, computer-aided brain diagnostic system (CABDS) for the detection of ASD could be very beneficial for autism analysis. The electroencephalogram (EEG) record of brain activity provides useful information regarding state. Hence, EEG signals are commonly used to detect brain diseases such as depression [17], epilepsy [18], schizophrenia [19], autism [20,21], and Parkinson’s disease [22].

## 2. Data Used

The instruments used to establish the pre-diagnosis criteria for ASD included the qualitative behavioral assessment by experts through internationally established descriptive standards, such as Childhood Autism Rating Scale (CARS), Autism Treatment Evaluation Checklist (ATEC), Psychoeducational Profile (PEP3), and Social Responsiveness Scale (SRS). Thereafter, EEG signals were acquired from 37 normal and 40 autistic children who ranged in age between 4 to 13 years. There were approximately 50% males and 50% females in each group. The autistic children were recruited from normal schools and centers of special education in Jordan. Informed consent was obtained from each parent prior to commencement of the study.

## 3. Methodology

### 3.1. Recording and Pre-Processing of Signals

**Figure 1:**
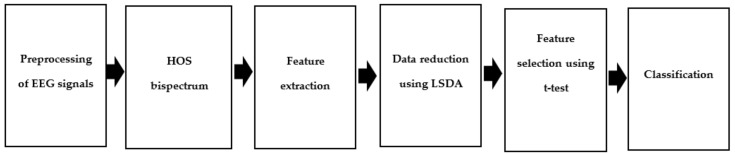
Workflow of our recommended method; * HOS: higher-order spectra; LSDA: locality sensitivity discriminant analysis.

Brain signals from the entire brain were recorded for 20 min as participants remained in the resting state. Each record had 64 channels of varying length, and the sampling frequency of each channel was 500 Hz. The frequency range considered was 0.3–40 Hz. All signals were discretized to 5519 samples in length. After segmentation, the higher-order spectra (HOS) bispectrum [23,24] is obtained. Nonlinear features are extracted from the HOS bispectrum plots. Figure 1 presents the proposed methodology.

### 3.2. HOS Bispectrum

The HOS bispectrum are obtained from the segmented ASD EEG signals. It is a nonlinear method which helps to provide the pase information present in the EEG signal.

### 3.3. Feature Extraction

Textural features are widely used in image analyses. These features allow images to be separated into regions of interest and classified thereafter. Textural features are exemplary as they capture crucial characteristics such as smoothness, consistency, and roughness of an image [25]. Textural parameters define the spatial distribution of intensity levels in a neighborhood. Some textural features extracted in image analyses include the co-occurrence matrix and difference-vector-based and run-length-matrix-based features. In this study, run-length-matrix-based features that were nonlinear were extracted after pre-processing. The features included the log energy, Kapoor entropy, max entropy, Rényi entropy [26], Shannon entropy [27], Vajda entropy [28], Yager entropy [29], short run emphasis [30], long run emphasis [31], gray-level nonuniformity [31], run length nonuniformity [31], run percentage [31], low gray-level run emphasis (LGRE) [32], high gray-level run emphasis (HGRE) [30], short run low gray-level run emphasis (SLGRE) [32], short run high gray-level run emphasis (SHGRE), long run low gray-level run emphasis (LLGRE) [30], and long run high gray-level run emphasis (LHGRE).

#### Description of Features

As EEG signals exhibit nonlinear characteristics, nonlinear features are used for classification of normal and anomalous signals [33]. Additionally, nonlinear features were used, as they are better able to capture complicated dynamic variants of EEG signals as compared to linear signals [34]. The short-run emphasis parameter increases when short runs take control in fine-grained image textures. Similarly, in long-run emphasis, the long runs take control in textures that are coarse or have sizeable uniform areas. Both short and long-run emphasis features describe the distribution of the corresponding long or short uniform runs in an image [35].

In LGRE, the feature metric increases as runs of low gray value govern the texture. Analogously, the measurement of HGRE spikes when the texture is controlled by large runs of gray value. Both low and high gray-level run emphasis features define the distribution of low or high gray-level runs within an image [32]. In gray-level nonuniformity, as gray level outliers dominate the histogram, the parameter increases, whereas in run length nonuniformity, the metric increases when the histogram is dominated by a few gray-level outliers. Both features explain the non-uniformity of the gray-levels or the length of the homogenous runs [32].

The run percentage feature details the homogeneousness of the histogram, and is at its peak when all runs are of uniform length regardless of gray-level [35]. In SLGRE, as more short runs of gray value dominate the texture, the metric of the feature increases. The measurement of the SLGRE increases as short runs with elevated intensity levels govern the texture. Both parameters generally describe the distribution of the short homogeneous runs with either high or low gray-levels [32]. As for LHGRE, it increases when long and high gray value runs are used together. The measurement of LLGRE increases as long runs with low gray-levels control the gray levels [35]. Both features define the distribution of long homogeneous runs with high or low gray-levels [32].

### 3.4. Feature Reduction and Selection

The extracted features are then subjected to locality sensitive discriminant analysis (LSDA) [36], a feature reduction technique. Data reduction techniques are employed to transform the features to a low-dimensional space for the discriminant analysis of data points [36]. LSDA works by determining the local manifold structure, and finding the prediction that maximizes the margin between data points from dissimilar classes at each local area. Unlike LSDA, other data reduction techniques such as principal component analysis (PCA) and linear discriminant analysis (LDA) do not determine the fundamental structure if the data appears to be proximal to the submanifold of the surrounding space; only the Euclidean structure is identified [36]. Being more advantageous, LSDA was thus used in this study. The reduced feature set was subjected to the independent *t*-test thereafter [37], in order to select the most significant features. Features with *p*-values ≥ 0.05 were discarded, whereas the remainder were used for classification.

### 3.5. Classification

A range of classifiers were explored in this study for the discrimination of classes. The LDA [38] describes Fisher’s linear discriminant in a basic way. It predicts by estimating the probability that a new set of input data fits each class. A prediction is made when the output data is formed with the class having the largest probability. Quadratic discriminant analysis (QDA) [39] which is an extension of LDA, was also used. It is based upon the supposition that the covariances are not certainly equal, and if they do happen to be equal, the decision boundary becomes linear, causing QDA to be reduced to LDA. The *k*-nearest neighbor (KNN) [40] classifier was also employed in this study. The feature classification takes place on the basis of the class that is most common to the feature’s *k*-nearest neighbors. Another classifier explored was the probabilistic neural network (PNN) classifier. PNN comprises layers wherein the concealed layer computes the probability density, whereas the summing layer combines the results. Support Vector Machine (SVM) has the ability to be generalized in a high-dimensional space, with a small training data size, and achieve high accuracy [41,42]. Hence, the SVM with radial basis function (SVM-RBF) kernel [43] and polynomial kernels [44] 1, 2, and 3 were also used. The RBF kernel is more adept than linear kernels due to its ability to nonlinearly map samples with nonlinear relationships into a higher dimensional space. The 10-fold cross-validation [45] technique was used to evaluate the performance of the classifiers.

## 4. Results

**Figure 2:**
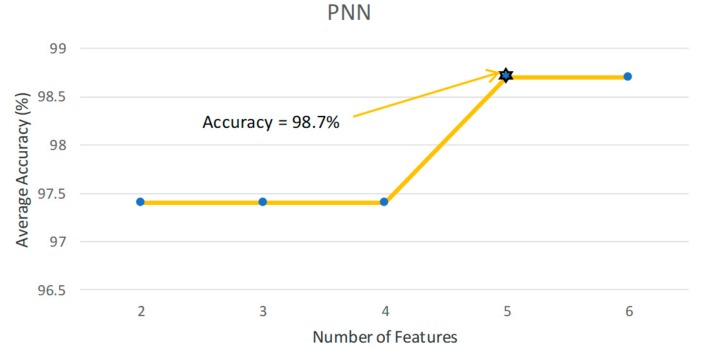
Classification accuracy versus number of features plot for the PNN model.

**Figure 3:**
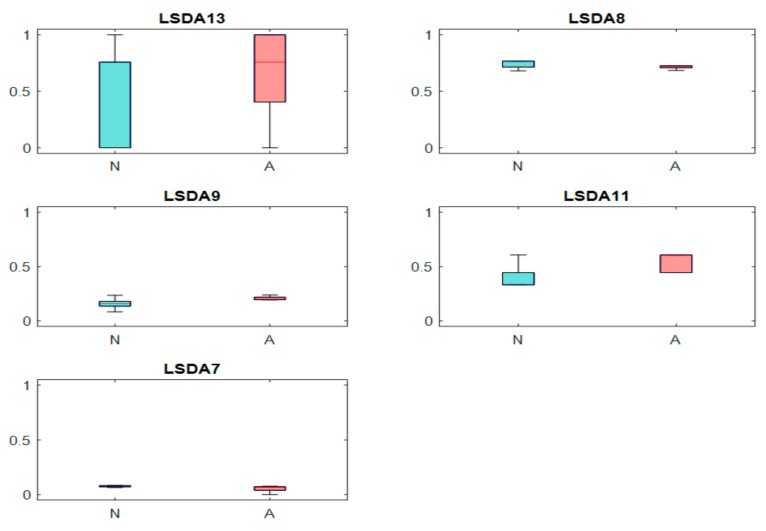
Boxplots of the top-performing locality sensitivity discriminant analysis (LSDA) features (N = normal, A = autism spectrum disorder (ASD)).

**Figure 4:**
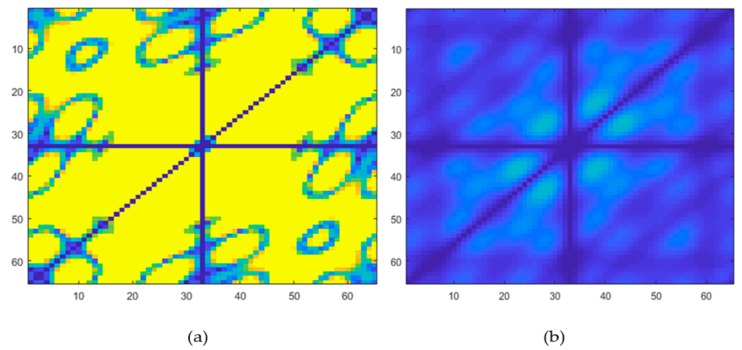
Bispectrum plots of (**a**) normal and (**b**) ASD classes (channel 64).

**Figure 5:**
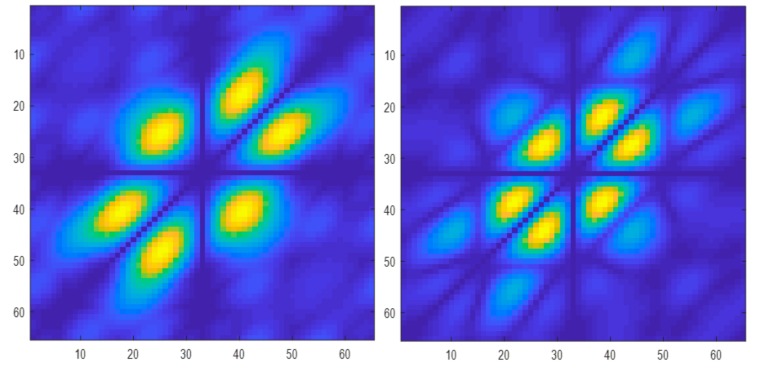
Bispectrum plots of (**a**) normal and (**b**) ASD classes (channel 10).

**Figure 6:**
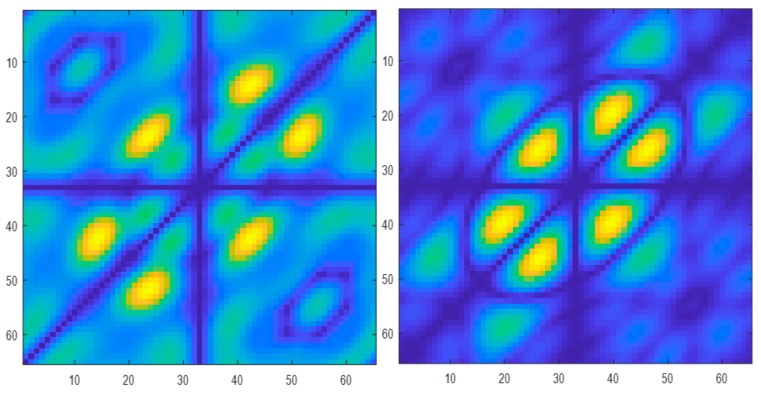
Bispectrum plots of (**a**) normal and (**b**) ASD classes (channel 50).

Table 1 presents the classification results based on the performance of the classifiers used. From the results obtained, it is evident that the PNN classifier achieved the highest accuracy, sensitivity, specificity, and positive predictive values of 98.70%, 100%, 97.30%, 97.56%, respectively, besting other classifiers. Table 2 presents the significant features selected using the *t*-test after LSDA feature reduction. As seen in Figure 2, only five features were needed to obtain the highest accuracy with the PNN model. Lower accuracies were obtained with the support vector machine with radial basis function (SVMRBF), k-NN, and SVM polynomial 3 classifiers, as lesser features are used to train the models. Hence, PNN is the most desirable classifier to be used to best differentiate ASD from normal EEG signals. Figure 3 presents the box plot of the top LSDA features. The boxplot was plotted by using the five most significant features, LSDA 13, LSDA 8, LSDA 9, LSDA 11, and LSDA 7, with *p* < 0.05, as seen in Table 2. It is observable that generally the mean of LSDA features was higher in the autism group than in the normal group. This could be due to higher variability in the autism class. Figure 4a,b shows the bispectrum plots of the normal and ASD classes, respectively, acquired from one channel (channel 64). More bispectrum plots of the normal and ASD classes for channels 10 and 50 are also shown in Figure 5a,b and Figure 6a,b, respectively. From these plots, it can be seen that the bispectrum patterns for the two classes were unique and distinct. Thus, the features used in our study had high discriminatory capacity.

## 5. Discussion

Table 3 summarizes prior studies in which a CABDS and EEG signals were used to assist in autism diagnosis. In the first study, the discrete wavelet transform (DWT) was employed to decompose acquired EEG signals. The signals were then mixed with artifacts and subjected to fast Independent Component Analysis (ICA) to obtain independent components. The signals were subsequently grouped into six different cases with different artifacts. The proposed method achieved an average correlation coefficient of 0.757 and regression of 0.699, demonstrating this to be an acceptable method for ASD detection [46].

DWT was also employed in the second study to decompose pre-processed EEG signals, thereby obtaining sub-bands. Entropy values were then computed on these bands to form the feature vector, which was put into an artificial neural network (ANN). Ten-fold validation was used for evaluation. The area under the receiver operating curve (ROC) with statistical measures obtained the highest accuracy of 99.7% for DWT coupled with Shannon entropy [47].

In the third study, a power spectral analysis was performed on pre-processed signals. The relative and absolute power were computed per frequency band, after which coherence indices were calculated for six intra-hemispheric and eight interhemispheric brain regions, respectively. Large differences in EEG power were reported between the groups, and larger EEG power in delta and theta power were found in the frontal and posterior regions [48].

Similarly, the wavelet transform was also employed in another study, in order to decompose the acquired EEG signals into six frequency bands, after which nonlinear features were extracted from these bands. The recursive feature elimination algorithm was used to select significant features, which were fed to a support vector machine with radial basis function (SVMRBF) classifier. High sensitivity and specificity values of nearly 100% were achieved for early detection of ASD [49].

Nonlinear features were extracted from time and frequency domains in the subsequent study, reporting that nonlinear features served as good indicators of early stages of ASD [50].

The spectral power and mean coherence parameters were computed from the EEG signals in another study. Student’s *t*-test was used to obtain the important differences for intragroup comparisons. It was reported that the spectral power of the theta rhythm was lower in autistic children than in healthy children, whereas the gamma power was larger [51].

In a separate study, variance in time and modified multiscale entropy features were extracted from pre-processed signals and fed to different classifiers. The highest accuracy of 79% was yielded with the naïve Bayes classifier [52].

In another study, the childhood autism rating scale coupled with statistical measures was used to examine the relationship between EEG anomalies and autism severity level. It was reported that the relationship between EEG anomalies and severity of autism was statistically significant [53].

After pre-processing the EEG signals, principal component analysis (PCA) was employed for dimensionality reduction, prior to extracting recurrence quantification analysis (RQA) nonlinear features from the signals, in another study. The SVM classifier coupled with leave-one-subject-out validation yielded a high classification accuracy of 92.9% [54].

Multiscale entropy (MSE) features were explored for the identification of ASD severity level in children, in another unique study. The MSE patterns that were obtained revealed that children with mild ASD had increased sample entropy values as compared to those with severe ASD. Also, the MSE values and physical representations were reported to represent children according to mild and severe ASD [55].

Elsewhere, EEG signals were extracted from children as they were subjected to images of different facial expressions (happiness, sadness, and calmness). A hybrid model was developed thereafter to map to the feature space. The mapping process was optimized and the resulting vector was input to the SVM classifier. The proposed method was able to discriminate normal versus ASD classes successfully [56].

In the next study, an artefact-free EEG segment was employed to calculate input values for successive analyses. The Implicit Function as Squashing Time (I-FAST) algorithm was employed subsequently for the selection of predictive parameters. The resulting invariant feature vector was then input to several classifiers, in which a highest accuracy of 92.8% was achieved with the random forest classifier coupled with leave-one-out cross-validation [57].

In another study, three different datasets were explored: eye, EEG, and a combination of both data. For each set, Fast Fourier Transform (FFT), entropy, and statistical features were extracted. PCA or sequential feature selection was used to obtain significant features, which were then input to different classifiers. The best performing models were naïve Bayes and logistic classifiers, which obtained an accuracy of 100% with the combination of eye and EEG data, whereas an accuracy of 100% was achieved with the logistic and deep neural network classifiers with only eye data [58].

In the next study, statistical features were extracted from the pre-processed EEG signals prior to and after the application of the discrete wavelet transform. Correlation-based feature selection was used thereafter to select significant features. The features were then input to various classifiers. A highest accuracy of 93% was achieved with the random forest classifier, using *k*-fold validation [59].

In the second-to-last study, the mean power spectral density of EEG features were computed after pre-processing. The features were then input to the SVM and artificial neural network (ANN) classifiers, and confusion matrixes were used to validate model performance. The highest accuracy of 90.5% (without emotions) was yielded by the ANN classifier for classification without emotions. A highest accuracy of 92.5% was also achieved with the ANN classifier, for classification with emotions [60].

Lastly, the global functional connectivity was computed after brain signals were acquired. Statistical analyses were conducted thereafter, and the results were supported by the autism diagnostic interview coupled with clinical evaluations. It was reported that the difference in global functional connectivity values between the high-risk (HR) and low-risk (LR) ASD groups and other groups in comparison was insignificant. In addition, the increase in the networks in the alpha range between the HR and LR groups and other groups by comparison was insignificant [61].

From Table 3, it is apparent that nonlinear features have been prevalently used to diagnose AD [49,50,54,55,57]. Additionally, SVM classifiers have also been commonly employed to classify EEG signals for the detection of ASD [52,54,56,58,59,60] similar to our study. Although a classification study was done, lower accuracies were achieved in the following studies: [52,54,57,59,60] as compared to ours. Although higher classification accuracies of 100% [58] and 99.71% [47] were achieved in these particular two studies as compared to our study, smaller data sizes were used for training in both studies. Although the results achieved in [30] are comparably high, the study reports on classification and correlation results, different from our study, which focused on classification alone. The remaining studies in Table 3 did not discuss classification; only correlation or comparison results were discussed. Hence, with the high accuracy obtained and larger data used as compared with most studies in Table 3, our proposed method is robust, as it has been tested on more data. There are several benefits and drawbacks of our technique:

Benefits:
  1. The recommended technique allows for rapid and accurate diagnosis of ASD.
  2. The diagnostic method is non-invasive.
  3. The method is promising, as the model used has been validated by 10-fold validation.
Drawbacks:
  1. Feature extraction and selection processes are done manually.
  2. This technique only supports a small data size; thus, sizeable data cannot be studied for early detection.

## 6. Summary

Both genetic and non-genetic factors may contribute to ASD. Disturbingly, its prevalence has been rising steadily over the past 20 years. Current diagnostics are either lengthy procedures, costly, or invasive, and exhibit other limitations. Hence, we have recommended a non-invasive and cost-effective CABDS to detect autism. After pre-processing, the EEG signals were converted to two-dimensional images using the HOS bispectrum. Nonlinear features were extracted thereafter, and the features were then reduced using LSDA. Student’s *t*-test was then employed to obtain significant features from the reduced feature set, which was input to various classifiers. A highest accuracy of 98.70% was yielded by the PNN classifier. Ten-fold validation was utilized to evaluate classifier performance. The robust system can potentially be used by healthcare professionals as a decision support tool for ASD detection.

## 7. Future work

In future work, we intend to gather a large volume of data over a period of a few years to utilize for the early detection of autism in children. Additionally, with the sizable data, we aim to use a deep learning model for classification [21,62,63,64,65]. When more data is used, the model can be trained well, and it is thus anticipated to perform well. Early detection of ASD assists patients as well as caregivers significantly in better managing the disorder.

## Figures and Tables

**Table 1:** Classification results of the various classifiers. KNN: *k*-nearest neighbor; SVMRBF: support vector machine with radial basis function; PNN: probabilistic neural network.

Classifier	Number of Features	Accuracy (%)	Sensitivity (%)	Specificity (%)	Positive Predictive Value (%)
Linear discriminant analysis	6	93.51	97.50	89.10	90.70
Quadratic discriminant analysis	5	85.71	87.50	83.78	85.37
SVM polynomial 1	6	93.51	97.50	89.19	90.70
SVM polynomial 2	5	97.40	97.50	97.30	97.50
SVM polynomial 3	4	96.10	95.00	97.30	97.44
KNN	3	92.21	92.50	91.90	92.50
SVMRBF	2	97.40	100.00	94.60	95.24
**PNN**	**5**	**98.70**	**100.00**	**97.30**	**97.56**

**Table 2:** Range (mean ± standard deviation) of features selected using *t*-test after linear discriminant analysis (LDA) feature reduction.

	Normal		ASD			
Features	Mean	SD	Mean	SD	*p*-Value	*t*-Value
LSDA13	−1756.04	1126.778	−801.964	1080.377	0.000309	3.786288
LSDA8	−1402.45	544.1245	−2004.56	909.222	0.000711	3.55602
LSDA9	−886.62	264.4797	−314.428	1157.47	0.003981	3.041854
LSDA11	1918.153	1133.604	2545.265	1297.72	0.026577	2.262406
LSDA7	−583.943	600.9221	−805.991	116.416	0.033149	2.209627
LSDA2	133.0712	364.5094	291.3328	311.3471	0.044995	2.040697
LSDA6	−833.493	651.3617	−998.316	145.0319	0.140299	1.505079
LSDA1	−385.252	98.16647	−548.472	803.8656	0.209993	1.273933
LSDA4	−531.886	140.8786	−567.485	125.164	0.246415	1.168582
LSDA5	−680.707	70.31738	−691.059	23.01104	0.397739	0.854162
LSDA14	−657.845	501.4798	−545.09	1308.884	0.614934	0.50615
LSDA21	−592.889	3.035538	−590.386	44.26157	0.723211	0.356711
LSDA10	796.1476	2058.705	922.609	867.6855	0.730657	0.346282
LSDA12	−5132.27	4467.789	−4754.77	5277.353	0.735127	0.339583
LSDA24	−1464.89	71.78779	−1461.35	7.848605	0.767	0.298501
LSDA23	−801.367	2047.65	−706.917	504.8907	0.786254	0.273003
LSDA28	1383.901	772.3631	1413.86	61.62772	0.815334	0.235248
LSDA27	1029.853	696.2088	1005.313	73.09999	0.832258	0.213319
LSDA29	585.8519	1.168018	585.4515	12.84125	0.845341	0.196346
LSDA22	−295.577	1400.244	−339.624	97.81121	0.849659	0.19091
LSDA17	445.7471	353.7109	485.0725	1609.695	0.880972	0.150629
LSDA15	460.2031	37.12207	463.1301	119.2549	0.883222	0.147686
LSDA19	−592.541	1998.428	−546.308	461.9218	0.891436	0.137369
LSDA20	−1035.72	1877.321	−993.439	381.1509	0.893739	0.134455
LSDA25	−588.542	1679.106	−621.33	116.2695	0.906315	0.118513
LSDA16	−1775.64	457.8857	−1799.15	1321.607	0.91614	0.105843
LSDA18	−1565.5	2122.529	−1523.15	1425.022	0.919109	0.101969
LSDA26	−663.813	14.99397	−664.267	26.89385	0.926769	0.092285
LSDA30	−653.938	158.0741	−653.208	37.69053	0.978273	0.027406
LSDA3	346.7321	107.2744	338.2296	2320.293	0.981649	0.023149

**Table 3:** A summary of studies using computer-aided brain diagnostic system (CABDS) for the prediction/diagnosis of ASD using electroencephalogram (EEG) signals.

Year Published	Techniques	Number of Participants/Database/Demographics	Results
[46] 2014	Discrete wavelet transform Artefact removal (fast ICA) Regression Correlation coefficient	Caltech, PhysioNet, and Swartz Center for Computational Neuroscience: 20 subjects	Average correlation coefficient: 0.7574 Regression: 0.6992
[50] 2014	Nonlinear features	N: 1 subject A: 1 patient	Nonlinear features can be used as pointers to diagnose at early stages of ASD.
[51] 2014	Spectral power Mean coherence Paired Student’s *t*-test	N: 24 subjects (boys; mean age of 6.05 ± 0.86 years) A: 27 patients (5.79 ± 1.42 years)	Spectral power of theta rhythm was lower in autistic children than in healthy children, whereas gamma power was larger.
[52] 2014	SVM Logistic regression Naïve Bayes	N: 30 subjects A : 19 patients	**Naïve Bayes**: **Ay**: **79%**
[48] 2015	Fourier power spectral examination Coherence indices	Child Psychiatry Outpatient Clinic: N: 21 subjects (aged between 4 and 12) A: 21 patients (aged between 4 and 12)	Statistically large differences in EEG power between the two groups; larger EEG power in delta and theta bands were found in the frontal and posterior regions.
[53] 2017	Independent *t*-test Pearson’s correlation coefficient Childhood autism rating scale	Psychiatric Outpatients Clinics, Faculty of Medicine N: 40 subjects (aged between 4 and 12) A : 40 patients (aged between 2 to 12 years, 28 boys)	Abnormal EEG signals and brainwave regions were found to correlate with ASD severity.
[47] 2017	Discrete wavelet transform Shannon entropy	King Abdulaziz University Brain Computer Interface Group: N: 10 subjects (males; aged 9 to 16) A: 9 patients (6 males, 3 females; aged 10–16)	**Discrete wavelet transform (DWT)+ Shannon entropy**: **Ay: 99.71%**
[56] 2017	Hybrid model SVM classifiers Optimisation of feature (KNN-Genetic algorithm)	N: 6 boys (aged 7 to 9 years) A: 6 children (4 boys, 2 girls; aged 7 to 9 years)	The method proposed is able to differentiate normal and ASD classes.
[57] 2017	I-FAST technique Leave-one-out cross- validation Multi-scale entropy Random forest classifier	Villa Santa Maria Institute N: 10 subjects (4 males, 6 females; aged 7 to 12 years) A: 15 patients (13 males, 2 females; aged 7 to 14 years)	**Random forest classifier**: **Ay: 92.8%**
[49] 2018	Wavelet transform Nonlinear features Statistical models ∙	Boston Children’s Hospital/Harvard Medical School N: 89 infants (with low risk of ASD) A: 99 infants (with older siblings having ASD diagnosis)	Sp, se: close to 100% Prediction scores correlated with actual scores.
[54] 2018	Recurrence quantification analysis features SVM classifier Principal component analysis Leave-one-subject-out, 10-fold validations	N: 7 subjects (aged 2–6 years) A: 7 patients (aged 2-6 years)	**SVM classifier**: **Ay**: **92.9%** **Se: 100%** **Sp: 85.7%**
[55] 2018	Averaged multiscale entropy Extraction of EEG signals related to facial expressions Multiscale entropy scale curve profiles	Mild A: 18 patients Severe A: 18 patients	Mean multiscale entropy (MSE) values were found to be higher in children with mild A as compared to those with severe A. Increased sample entropy values in children with mild A.
[60] 2018	SVM, artificial neural network classifiers Power spectral density Emotions, EEG signals Confusion matrixes	-	**Classification of ASD versus normal without emotions**: Artificial neural network: **Ay: 90.5%** C**lassification of ASD versus normal with emotions**: Artificial neural network: **Ay: 92.5%** Autistic children express a more complexed emotion than normal children.
[58] 2019	Eye movements coupled with EEG SVM, logistic, deep neural network, naïve Bayes classifiers Statistical, entropy, FFT values 10 × 2 cross-validation	34 participants	**Eye + EEG data:** Naïve Bayes: **Ay: 100%** Logistic: **Ay: 100%** Only eye data: Logistic: **Ay: 100%** Deep neural network: **Ay: 100%**
[59] 2019	Discrete wavelet transform Correlation-based feature selection Logistic, SVM, naïve Bayes, random forest classifiers *k*-fold cross validation	N: 5 subjectsA: 10 patients (9 males, 6 females; between 5 and 17 years)	**Random forest classifie**r: **Ay: 93%**
[61] 2019	Global functional connectivity Shapiro–Wilk test, Levene’s test Network-based statistics	N (low risk infants): 20 subjects A (high-risk infants): 81 patients	Insignificant increase in global functional connectivity and networks in the alpha range between high-risk (HR) and low-risk (LR) groups and other groups being compared.
**Present study**	**Texture parameters** **Local sensitivity discriminant analysis** ***t*-test** **10-fold cross validation** **PNN classifier**	N: 37 healthy A: 40 patients	**Probabilistic neural network classifier**: **Ay: 98.7%**

* N: normal, A: ASD, Ay: accuracy, Se: sensitivity, Sp: specificity.

## References

[1] Kim D.G., Park H.R., Lee J.M., Moon H.E., Lee D.S., Kim B.N., Kim J., Paek S.H. (2016). A short review on the current understanding of autism spectrum disorders. Exp. Neurobiol..

[2] Gharani N., Benayed R., Mancuso V., Brzustowicz L.M., Millonig J.H. (2004). Association of the homeobox transcription factor, ENGRAILED 2, 3, with autism spectrum disorder. Mol. Psychiatry.

[3] Miles J.H. (2011). Autism spectrum disorders-A genetics review. Genet. Med..

[4] Kern J.K., Jones A.M. (2007). Evidence of toxicity, oxidative stress, and neuronal insult in autism. J. Toxicol. Environ. Health B Crit. Rev..

[5] Kolevzon A.R., Raz Gross A. (2007). Prenatal and perinatal risk factors for autism. Arch. Pediatrics Adolesc. Med..

[6] NIH Autism Spectrum Disorder Fact Sheet. https://www.ninds.nih.gov/Disorders/Patient-Caregiver-Education/Fact-Sheets/Autism-Spectrum-Disorder-Fact-Sheet.

[7] Fisch G.S. (2013). Erratum to In the article by Gene S. Fisch, entitled “Nosology and Epidemiology in Autism: Classification Counts” in. American Journal of Medical Genetics Part C. Am. J. Med. Genet. Part A.

[8] Robinson E.B., Lichtenstein P., Anckarsäter H., Happé F., Ronald A. (2013). Examining and interpreting the female protective effect against autistic behavior. Proc. Natl. Acad. Sci. USA.

[9] Mattila M.L., Kielinen M., Linna S.L., Jussila K., Ebeling H., Bloigu R., Joseph R.M., Moilanen I. (2011). Autism spectrum disorders according to DSM-IV-TR and comparison with DSM-5 draft criteria: An epidemiological study. J. Am. Acad. Child Adolesc. Psychiatry.

[10] Leventhal B.L., Kim Y.S., Koh Y.J., Fombonne E., Laska E., Lim E.C., Cheon K.A., Kim S.J., Kim Y.K., Lee H. (2011). Prevalence of autism spectrum disorder in a total population sample. Am. J. Psychiatry.

[11] Falkmer T., Anderson K., Falkmer M., Horlin C. (2013). Diagnostic procedures in autism spectrum disorders: A systematic literature review. Eur. Child Adolesc. Psychiatry.

[12] Zalla T., Sperduti M. (2013). The Amygdala and the Relevance Detection Theory of Autism: An Evolutionary Perspective. Front. Hum. Neurosci..

[13] Howard M.A., Cowell P.E., Boucher J., Broks P., Mayes A., Farrant A., Roberts N. (2000). Convergent neuroanatomical and behavioural evidence of an amygdala hypothesis of autism. Neuroreport.

[14] Abell F., Krams M., Ashburner J., Passingham R., Friston K., Frackowiak R., Happe F., Frith C., Frith U. (2013). The neuroanatomy of autism: A voxel-based whole brain analysis of structural scans. Sci. Ment. Heal. Vol. 2 Autism.

[15] Nam D., Barrack R.L., Potter H.G. (2014). What are the advantages and disadvantages of imaging modalities to diagnose wear-related corrosion problems?. Clin. Orthop. Relat. Res..

[16] Salmon E., Bernard Ir C., Hustinx C. (2015). Pitfalls and limitations of PET/CT in brain imaging. Semin. Nucl. Med..

[17] Acharya U.R., Oh S.L., Hagiwara Y., Tan J.H., Adeli H., Subha D.P. (2018). Automated EEG-based screening of depression using deep convolutional neural network. Comput. Methods Programs Biomed..

[18] Acharya U.R. (2019). Characterization of focal EEG signals: A review. Futur. Gener. Comput. Syst..

[19] Jahmunah V., Oh S.L., Rajinikanth V., Ciaccio E.J., Cheong K.H., Arunkumar N., Acharya U.R. (2019). Automated detection of schizophrenia using nonlinear signal processing methods. Artif. Intell. Med..

[20] Bhat S., Acharya U.R., Adeli H., Bairy G.M., Adeli A. (2014). Autism: Cause factors, early diagnosis and therapies. Rev. Neurosci..

[21] Hadoush H., Alafeef M., Abdulhay E. (2019). Automated identification for autism severity level: EEG analysis using empirical mode decomposition and second order difference plot. Behav. Brain Res..

[22] Oh S.L., Hagiwara Y., Raghavendra U., Yuvaraj R., Arunkumar N., Murugappan M., Acharya U.R. (2018). A deep learning approach for Parkinson’s disease diagnosis from EEG signals. Neural Comput. Appl..

[23] Collis W.B., White P.R., Hammond J.K. (1998). Higher-Order Spectra: The Bispectrum and Trispectrum. Mech. Syst. Signal Process..

[24] Acharya U.R., Vidya K.S., Koh J.E.W., Martis R.J., Tan J.H., Oh S.L., Adam M., Hagiwara Y., Mookiah M.R.K., Chua K.P. (2017). Application of higher-order spectra for the characterization of coronary artery disease using electrocardiogram signals. Biomed. Signal Process. Control.

[25] Tan J.H., Ng E.Y.K., Acharya U.R., Chee C. (2010). Study of normal ocular thermogram using textural parameters. Infrared Phys. Technol..

[26] Savare G., Toscani G. (2014). The concavity of rényi entropy power. IEEE Trans. Inf. Theory.

[27] Shannon C.E. (1948). A mathematical theory of communication. Bell Syst. Tech. J..

[28] Darbellay G.A., Vajda I. (2000). Entropy expressions for multivariate continuous distributions. IEEE Trans. Inf. Theory.

[29] Hu Q., Yu D. (2004). Entropies of fuzzy indiscernibility relation and its operations. Int. J. Uncertain. Fuzziness Knowlege-Based Syst..

[30] Tang X. (1998). Automated diagnosis of glaucoma using texture and higher order spectra features. IEEE Trans. Image Process..

[31] Galloway M.M. (1975). Texture analysis using gray level run lengths. Comput. Graph. Image Process..

[32] Xu D.H., Kurani A.S., Furst J.D., Raicu D.S. (2004). Run-length encoding for volumetric texture. Proc. Fourth IASTED Int. Conf. Vis. Imaging Image Process.

[33] Hornero R., Abasolo D., Jimeno N., Sanchez C.I., Poza J., Aboy M. (2006). Variability, regularity, and complexity of time series generated by schizophrenic patients and control subjects. IEEE Trans. Biomed. Eng..

[34] Acharya U.R., Sudarshan V.K., Adeli H., Santhosh J., Koh J.E.W., Adeli A. (2015). Computer-aided diagnosis of depression using EEG signals. Eur. Neurol..

[35] Haidekker M. (2011). Advanced Biomedical Image Analysis.

[36] Cai D., He X., Zhou K., Han J., Bao H. (2007). Locality Sensitive Discriminant Analysis. Proceedings of the 20th International Joint Conference on Artificial Intelligence.

[37] Kim T.K. (2015). T test as a parametric statistic. Korean J. Anesthesiol..

[38] Hasan M.R., Ibrahimy M.I., Motakabber S.M.A., Shahid S. (2015). Classification of multichannel EEG signal by linear discriminant analysis. Progress in Systems Engineering.

[39] Ghojogh B., Crowley M. (2019). Introduction to machine learning: K-nearest neighbors. Ann. Transl. Med..

[40] Zhang Z. (2016). Support vector machines. Ann. Transl. Med..

[41] Bakheet S. (2017). An SVM framework for malignant melanoma detection based on optimized HOG features. Computation.

[42] Men S., Yan L., Liu J., Qian H., Luo Q. (2017). A classification method for seed viability assessment with infrared thermography. Sensors.

[43] Apostolidis-Afentoulis V., Lioufi K.I. SVM Classification with Linear and RBF Kernels. https://www.researchgate.net/publication/279913074_SVM_Classification_with_Linear_and_RBF_kernels.

[44] Karatzoglou A., Meyer D., Hornik K. (2006). Support vector machines in R. J. Stat. Softw..

[45] Duda D.G.S.R.O., Hart P.E. (2001). Pattern Classification.

[46] Jadhav P.N., Shanamugan D., Chourasia A., Ghole A.R., Acharyya A., Naik G. Automated detection and correction of eye blink and muscular artefacts in EEG signal for analysis of Autism Spectrum Disorder. Proceedings of the 36th Annual International Conference of the IEEE Engineering in Medicine and Biology Society.

[47] Djemal R., Al Sharabi K., Ibrahim S., Alsuwailem A. (2017). EEG-Based computer aided diagnosis of autism spectrum disorder using wavelet, entropy, and ANN. Biomed. Res. Int..

[48] Elhabashy H., Raafat O., Afifi L., Raafat H., Abdullah K. (2015). Quantitative EEG in autistic children. Egypt. J. Neurol. Psychiatry Neurosurg..

[49] Bosl W.J., Tager-Flusberg H., Nelson C.A. (2018). EEG analytics for early detection of autism spectrum disorder: A data-driven approach. Sci. Rep..

[50] Bhat S., Acharya U.R., Adeli H., Bairy G.M., Adeli A. (2014). Automated diagnosis of autism: In search of a mathematical marker. Rev. Neurosci..

[51] Lushchekina E.A., Podreznaya E.D., Lushchekin V.S., Novototskii-Vlasov V.Y., Strelets V.B. (2014). Comparative studies of EEG theta and gamma rhythms in normal children and children with early childhood autism. Neurosci. Behav. Physiol..

[52] Eldridge J., Lane A.E., Belkin M., Dennis S. (2014). Robust features for the automatic identification of autism spectrum disorder in children. J. Neurodev. Disord..

[53] Yousef A., Youssef U., El-Shabrawy A., Abdel Fattah N.A., Khedr H., Khedr H. (2017). EEG abnormalities and severity of symptoms in non-epileptic autistic children. Egypt. J. Psychiatry.

[54] Heunis T., Aldrich C., Peters J.M., Jeste S.S., Sahin M., Scheffer C., Vries P.J. (2018). Recurrence quantification analysis of resting state EEG signals in autism spectrum disorder—A systematic methodological exploration of technical and demographic confounders in the search for biomarkers. BMC Med..

[55] Hadoush H., Alafeef M., Abdulhay E. (2019). Brain complexity in children with mild and severe autism spectrum disorders: Analysis of multiscale entropy in EEG. Brain Topogr..

[56] Hashemian H.P.M. (2017). Decision-level fusion-based structure of autism diagnosis uisng interpretation of EEG signals related to facial expression modes. Neurophysiology.

[57] Grossi E., Olivieri C., Buscema M. (2017). Diagnosis of autism through EEG processed by advanced computational algorithms: A pilot study. Comput. Methods Programs Biomed..

[58] Thapaliya S., Jayarathna S., Jaime M. (2018). Evaluating the EEG and eye movements for autism spectrum disorder. Proceedings of the 2018 IEEE International Conference on Big Data.

[59] Haputhanthri D., Brihadiswaran G., Gunathilaka S., Meedeniya D., Jayawardena Y., Jayarathna S., Jaime M. (2019). An EEG based channel optimized classification approach for autism spectrum disorder. Proceedings of 2019 Moratuwa Engineering Research Conference (MERCon).

[60] Harun N.H., Hamzah N., Zaini N., Sani M.M., Norhazman H., Yassin I.M. (2018). EEG classification analysis for diagnosing autism spectrum disorder based on emotions. J. Telecommun. Electron. Comput. Eng..

[61] Haartsen R., Jones E.J.H., Orekhova E.V., Charman T., Johnson M.H. (2019). Functional EEG connectivity in infants associates with later restricted and repetitive behaviours in autism: A replication study. Transl. Psychiatry.

[62] Acharya U.R., Fujita H., Oh S.L., Raghavendra U., Tan J.H., Adam M., Gertych A., Hagiwara Y. (2018). Automated identification of shockable and non-shockable life-threatening ventricular arrhythmias using convolutional neural network. Future Gener. Comp. Syst..

[63] Oh S.L., Ng E.Y.K., San Tan R., Acharya U.R. (2018). Automated diagnosis of arrhythmia using combination of CNN and LSTM techniques with variable length heart beats. Comput. Biol. Med..

[64] Raghavendra U., Fujita H., Bhandary S.V., Gudigar A., Tan J.H., Acharya U.R. (2018). Deep convolution neural network for accurate diagnosis of glaucoma using digital fundus images. Inf. Sci..

[65] Tan J.H., Hagiwara Y., Pang W., Lim I., Oh S.L., Adam M., Tan R.S., Chen M., Acharya U.R. (2018). Application of stacked convolutional and long short-term memory network for accurate identification of CAD ECG signals. Comput. Biol. Med..

